# Native T1-mapping displays the extent and non-ischemic patterns of injury in acute myocarditis without the need for contrast agents

**DOI:** 10.1186/1532-429X-16-S1-O6

**Published:** 2014-01-16

**Authors:** Vanessa M Ferreira, Stefan K Piechnik, Erica Dall'Armellina, Theodoros D Karamitsos, Jane M Francis, Ntobeko A Ntusi, Cameron Holloway, Robin Choudhury, Attila Kardos, Matthew D Robson, Matthias G Friedrich, Stefan Neubauer

**Affiliations:** 1 Division of Cardiovascular Medicine, Radcliffe Department of Medicine, University of Oxford, Oxford, UK; 2 Department of Cardiology, Milton Keynes NHS Hospital Foundation trust, Milton Keynes, UK; 3 Department of Cardiology, Université de Montréal, Montréal, Quebec, Canada; 4 Stephenson Cardiovascular MR Centre, Libin Cardiovascular Institute of Alberta, University of Calgary, Calgary, Alberta, Canada

## Background

Acute myocarditis is typically diagnosed on CMR using multiple techniques, including late gadolinium enhancement (LGE) imaging, which require contrast administration. T1-mapping is significantly more sensitive than conventional T2-weighted (T2W) and LGE imaging in detecting myocarditis, without the need for contrast agents.

## Methods

**Figure 1:**
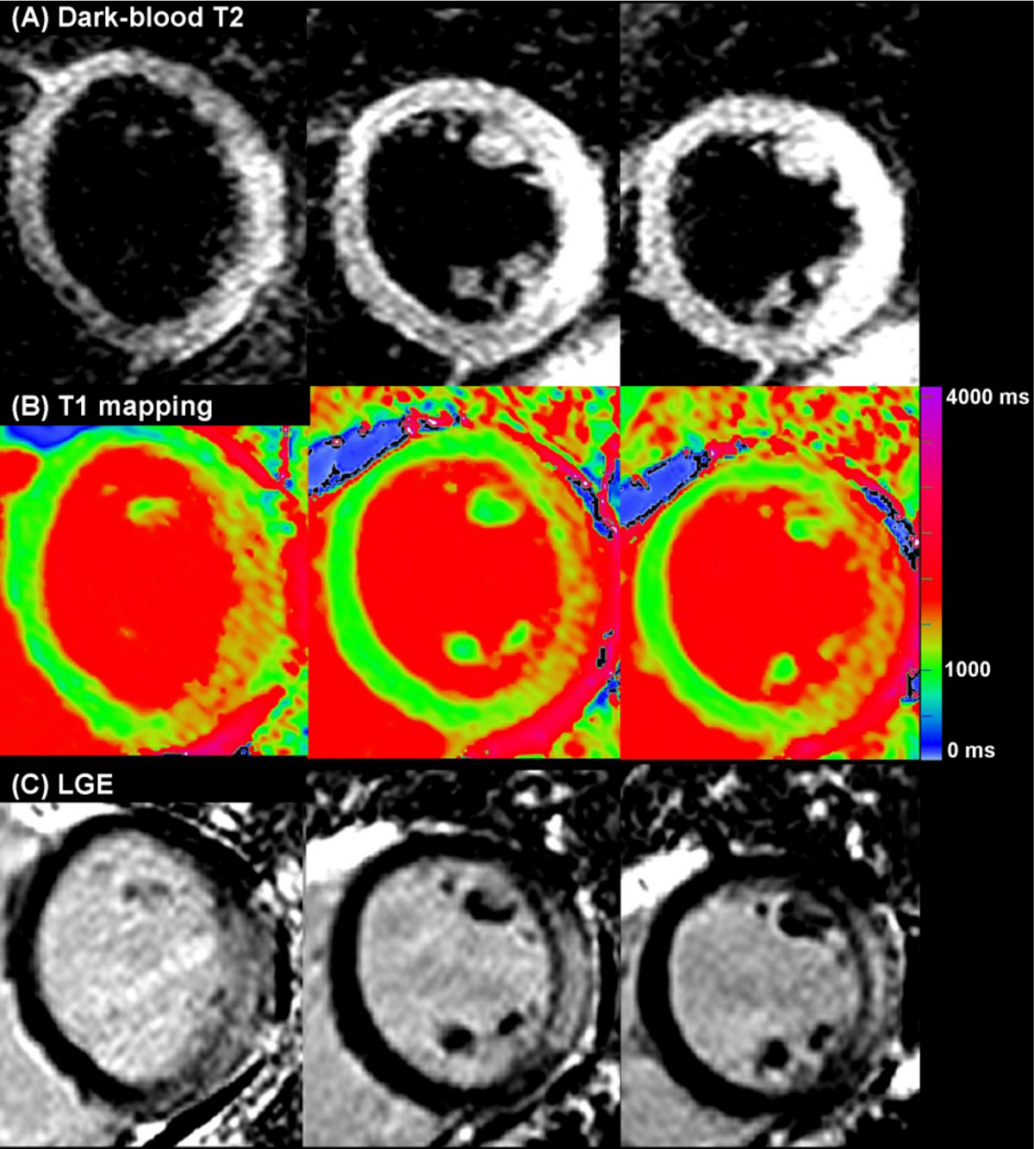
**CMR tissue characterization using (A) dark-blood T2-weighted imaging; (B) native T1-mapping (ShMOLLI); (C) late gadolinium enhancement imaging**.

We investigated 60 patients with suspected acute myocarditis (median 3 days from presentation) and 50 controls using CMR (1.5T), including: (1) dark-blood T2W imaging; (2) T1-mapping (ShMOLLI); (3) LGE (Figure 1). Analysis included: (1) global myocardial T2 signal intensity (SI) compared to skeletal muscle; (2) myocardial T1 times; (3) areas of injury by T2W, T1-mapping and LGE.

## Results

**Figure 2:**
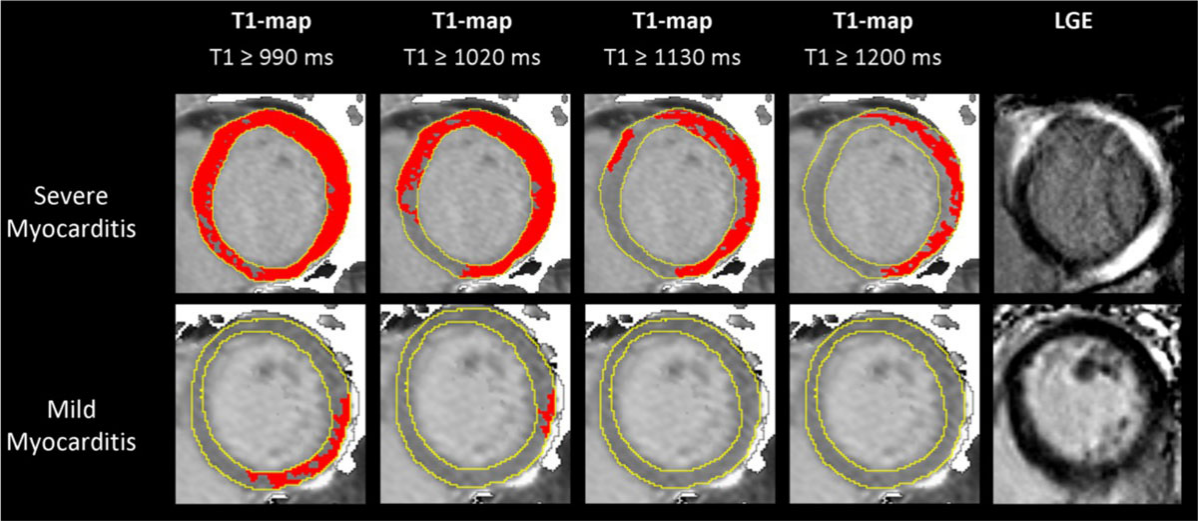
**Native T1-maps displaying the non-ischemic patterns of injury in acute myocarditis using incremental T1 thresholds**.

Compared to controls, patients had significantly more edema (global myocardial T2 SI ratio 1.71 ± 0.27 vs.1.56 ± 0.15), higher mean myocardial T1 (1011 ± 64 ms vs. 946 ± 23 ms) and more areas of injury measured by T2 (median 5% vs. 0%), T1 (median 32% vs. 0.7%) and LGE (median 11% vs. 0%); all p < 0.001. A threshold of T1 > 990 ms (sensitivity 90%, specificity 91%) detected significantly larger areas of involvement than T2W and LGE imaging in patients, and additional areas of injury when T2W and LGE were negative. Using incremental thresholds, T1-mapping can display the non-ischemic patterns of injury typical of myocarditis (Figure 2).

## Conclusions

In acute myocarditis, native T1-mapping can display the typical non-ischemic patterns similar to LGE imaging without the need for contrast agents. T1-mapping also detects additional areas of involvement and identifies extra cases beyond T2W and LGE imaging.

## Funding

This study is funded by the Oxford National Institute for Health Research Biomedical Research Centre Programme. VMF received funding from the Alberta Innovates Health Solutions (AIHS) Clinical Fellowship and the University of Oxford Clarendon Fund Scholarship. RC is a Wellcome Trust Senior Research Fellow in Clinical Science. SN and RC acknowledge support from the British Heart Foundation Centre of Research Excellence, Oxford.

